# Evaluating the frequency of neurological symptoms in COVID‐19 patients: A cross‐sectional study

**DOI:** 10.1002/hsr2.1400

**Published:** 2023-07-21

**Authors:** Shima Rasouli, Payam Emami, Farhad Azadmehr, Farzaneh Karimyan

**Affiliations:** ^1^ Student Research Committee Kurdistan University of Medical Sciences Sanandaj Iran; ^2^ Department of Emergency Medical Sciences, Faculty of Paramedical sciences Kurdistan University of Medical Sciences Sanandaj Iran; ^3^ MSc in Nursing Education, Faculty Member of Boukan Nursing Faculty Urmia University of Medical Sciences Urmia Iran; ^4^ Associate Professor of Neurology Kurdistan University of Medical Sciences Sanandaj Iran

**Keywords:** central nervous system, COVID‐19, peripheral nervous system

## Abstract

**Background and Aims:**

Due to the recent emergence of COVID‐19, the exact pathology of this disease has not been determined. Therefore, this study evaluated the frequency of neurological symptoms in patients with COVID‐19.

**Methods:**

This cross‐sectional study was conducted on 2200 in patients with COVID‐19 who were selected from an educational hospital in Sanandaj, Iran, from April 2020 to March 2021. The research samples were selected by census, all patients with COVID‐19 were admitted to the hospital. The data collection tool was a checklist of the studied variables (dizziness, headache, and impaired consciousness) prepared by the researchers based on the specialists' opinions. The researcher completed these checklists based on the patients' hospitalization records. The data were analyzed by descriptive and analytical statistical tests using SPSS Software Version 20. The quantitative variables were compared using the independent *t*‐test. The *χ* ^2^ test was also used to compare qualitative variables. A *p* Value of less than 0.05 was considered statistically significant.

**Results:**

The mean age of the patients was 57.41 years old, of whom 53.1% were male. The average blood oxygen level of the patients was 88.10%, and most disease symptoms were related to shortness of breath and cough, with a frequency of 24.3%. In addition, 20.8% of patients needed hospitalization in intensive care unit. The highest frequency of central and peripheral nervous system manifestations was related to headache, ageusia (loss of sense of taste), hyposmia (A decreased sense of smell and anosmia (The complete loss of smell). Finally, 15.3% of patients died, and 84.7% recovered. The analytical findings showed a significant relationship between the disease outcome and patients' dizziness, consciousness disorder, seizure and ageusia. There was a significant relationship between gender and headache in patients. There was a significant difference between the mean age and oxygen level with central and peripheral nervous system manifestations (dizziness, headache, impaired consciousness, smell disorder) and the disease outcome in patients.

**Conclusion:**

The pathophysiology of COVID‐19 virus infection involving the central nervous system is not fully understood. Neurological symptoms of this virus include delirium, headache, decreased level of consciousness, and seizures. Identifying the symptoms and mechanisms of neurological complications of COVID‐19 is necessary for proper screening and complete treatment because a patient infected by COVID‐19 may not show respiratory failure signs but may be a carrier. A complete and accurate knowledge of the symptoms and complications of this infection for proper screening of patients to prevent transmission and spread of this disease is critically needed.

## 1 INTRODUCTION

Coronaviruses are essential pathogens in human and animal. A new coronavirus (severe acute respiratory syndrome coronavirus 2 [SARS‐CoV‐2]) became recognized in 2019 for the first time in Wuhan, China, as the cause of pneumonia in people.^1^, ^2^, ^3^, ^4^ The disease caused by this virus was named COVID‐19 by the World Health Organization,^5^ spread throughout China and other countries, and became a critical health condition worldwide. The average incubation period of the SARS‐CoV‐2 virus is 3−7 days.^6^, ^7^

Coronaviruses are a large family of viruses, causing diseases from simple colds to severe health problems, such as Middle East Respiratory Syndrome (MERS‐COV) and Severe Respiratory Syndrome (SARS).^5^ COVID‐19 is a new strain of this virus family, which was first introduced in China and never observed in human populations earlier before.^7^, ^8^ Different clinical symptoms have been reported for COVID‐19, from asymptomatic to severe forms of the disease, leading to acute respiratory distress (ARDS) and requiring special care, mechanical ventilation, and multiple organ failure.^6^ Common symptoms of this disease include fever, dizziness, fatigue, nasal congestion, and muscle pain. Standard tests include lymphopenia, high levels of C‐reactive protein in plasma, and increased lactate dehydrogenase. About 7−10% of patients progress critically, and 1−2% die, even though these results vary by region.^9^

This disease is transmitted by spreading tiny respiratory droplets of infected people or touching the face after hand contact with contaminated surfaces. Although most affected people initially have mild symptoms similar to a simple cold, more severe symptoms appear a few days after affecting.^10^ These symptoms include a non‐productive cough, dizziness, sore throat, fever, shortness of breath, muscle pain, and loss of sense of smell. Gastrointestinal symptoms such as diarrhea, skin complications, and red eyes have also been observed in some patients.^11^ Failure in the functioning of the central nervous system, heart, kidney, and liver is observed in some patients with COVID‐19.^12^

Studies have shown that the COVID‐19 virus can induce fever, nonproductive cough, shortness of breath, myalgia, fatigue, malaise, and gastrointestinal disturbances in affected patients. In more severe cases, infections cause viral pneumonia, severe ARDS, and even death.^13^, ^14^

In addition to respiratory failure and cardiac symptoms, this disease can also cause neurological symptoms in patients, including headaches, dizziness, ataxic encephalitis, stroke, and seizures affecting the central nervous system.^2^, ^15^ Neurological complications include demyelinating conditions after infection.^16^ The most commonly reported symptoms include headache and dizziness, encephalopathy (abnormal brain function or brain structure), delirium, blurred vision, tinnitus (ringing in the ears) and fatigue.^4^ The mentioned difficulties are cerebrovascular accidents such as cerebral ischemic stroke, intracerebral hemorrhages, and cerebral vasculitis.^2^, ^11^ The peripheral nervous system might also be affected. Ageusia, anosmia/hyposmia, myalgia, paresthesia, encephalitis (inflammation of the active tissues of the brain) or encephalopathy and Guillain‐Barré syndrome (A disorder in which your body's immune system attacks your nerves) are the most reported disorders affecting the peripheral nervous system in patients with COVID‐19.^11^, ^17^, ^18^ Given the limited studies regarding COVID‐19 pathology and its pandemic in 2020, many of the disease symptoms are unknown and lack a definitive treatment. Therefore, this study investigated the neurological manifestations of COVID‐19 patients treated at educational hospitals in Sanandaj, Iran, during the pandemic wave to understand the wide range of neurological symptom manifestations in their global occurrence.

## 2 METHOD

This cross‐sectional study was conducted on all COVID‐19 patients hospitalized between April 2020 to March 2021. The sampling was performed by the census, and 2200 hospitalized patients were included in the study. The minimum required sample size was calculated using the online sample size calculator (the estimated COVID‐19 prevalence of 33%,^19^ type 1 error rate (*α*) of 0.05, and absolute error of precision (*d*) of 0.02); and it was found to be 2124 (https://riskcalc.org/samplesize/). To find the final adjusted sample size, allowing nonresponse rate of 10%, the adjusted sample size was 2336. The data of patients were collected by the researcher after obtaining permission from the ethics committee.

The files of hospitalized patients in medical records were used to collect information. The relevant units in the university and hospital were coordinated to obtain permission to access the patients' files. In addition, a picture archiving and communication system (PACS) was used to access CT scans of patients' brains. Medical records were reviewed only after verbal consent was obtained from patients who had been discharged by the phone number listed in their records, and this consent was waived for patients who had died. All patients whose files have been declared positive for COVID‐19 by the relevant specialists were reviewed. Then, all patients with neurological symptoms were examined simultaneously by neurological specialists in terms of symptoms and diagnostic procedures. Due to the observational nature of the study design, neither patients nor investigators could be blinded. To reduce the chances of biasing the result, data analyzer and outcome assessor were blinded. Patients were never contacted during the entire duration of the study because of the inherent nature of the observational research.

## 3 DATA COLLECTION TOOLS

A checklist of studied variables, including age, gender, chief complaint, need for intensive care unit (ICU)* admission, neurologist visit, oxygen saturation (SpO_2_), central nervous system manifestations (dizziness, headache, impaired consciousness, focal neurological symptoms, stroke, intracerebral hemorrhage, ataxia, and seizures), and peripheral nervous system manifestations (taste, smell and vision abnormalities, myopathy, and polyneuropathy) was designed for COVID‐19 patients by researchers in the clinical faculty of the neurology department. Neurological manifestations were later confirmed by a thorough review of all available patient records by two researchers using hospital's health informative system (HIS) via checklists.

## 4 INCLUSION AND EXCLUSION CRITERIA

The study's inclusion criteria were positive and hospitalized COVID‐19 patients, and the exclusion criteria included an underlying neurological disease.

## 5 DATA ANALYSIS METHOD

The data collected in this study were analyzed using SPSS software version 20 after the information was collected. The quantitative data were described using mean and SD, and the qualitative data were assessed by frequency and percentage. Then, the quantitative variables were compared using the independent *t*‐test. The *χ* ^2^ test was also used to compare qualitative variables. A *p* value of less than 0.05 was considered statistically significant.

## 6 RESULTS

The mean age of the patients was 57.41 years old, of whom 53.1% were male. The average blood oxygen level of the patients was 88.10%, and most disease symptoms were related to shortness of breath and cough (24.3%). In addition, 20.8% of patients needed hospitalization in ICU. The highest frequency of central and peripheral nervous system manifestations was related to headache, ageusia, hyposmia and anosmia. In addition, 2.5% of the studied patients were visited by a neurologist. Finally, 15.3% of patients died, and 84.7% recovered (Tables 1, 2, 3, 4).

**Table 1:** Average quantitative variables in the studied patients.

Variable	Quantity	Mean	SD	Min	Max
Age	2200	57.41	16.72	16	99
Blood oxygen level	2200	88.10	8.81	35	100

**Table 2:** The frequency distribution of qualitative variables.

Variable		Frequency	Percentage
Gender	Male	1169	53.1
	Female	1031	46.9
Disease consequences	Recovery	1864	84.7
	Death	336	15.3
Patient visit by the neurologist	Yes	55	2.5
	No	2145	97.5
Need to be admitted to the ICU	Yes	457	20.8
	No	1743	79.2

Abbreviation: ICU, intensive care unit.

**Table 3:** The distribution of the frequency of early disease symptoms.

Variables		Frequency	Percentage
Early disease symptoms	Age	114	5.2
	Shortness of breath	329	15
	Weakness, lethargy, and loss of appetite	371	16.9
	Headache	30	1.4
	Cough and dry cough	125	5.7
	Myalgia and shortness of breath	103	4.7
	Fever and chills + shortness of breath + Myalgia	20	0.9
	Restlessness	11	0.5
	Shortness of breath + cough	534	24.3
	Fever and chills + shortness of breath	96	4.4
	Headache + shortness of breath + Myalgia	111	5
	Shortness of breath + weakness, lethargy, and loss of appetite	197	9
	Nausea, vomiting, and abdominal pain	69	3.1
	More than four signs	90	4.1
	Total	2200	100

**Table 4:** The distribution of central and peripheral nervous system manifestations.

Variable		Frequency	Percentage
Manifestations of the central nervous system	Dizziness	43	2
	Headache	149	6.8
	Impaired consciousness	53	2.4
	Focal neurological symptoms	6	0.03
	stroke	3	0.1
	Intracerebral hemorrhage	0	0
	Ataxia	5	0.2
	Convulsions	7	0.3
Manifestations of the peripheral nervous system		46	2.1
	Hyposmia/Anosmia	80	3.6
	Ageusia	46	2.09
	Visual impairment	3	0.1
	Polyneuropathy	4	0.2
	Myopathy	1	0.001

Table 5 presents the relationship between the disease outcome and manifesting the central nervous system. Chi‐square analysis shows a statistically significant relationship between the disease outcome and dizziness, impaired consciousness, and seizures in the studied patients. A correlation is also shown between the disease outcome and the peripheral nervous system manifestations in the study patients. There is a significant relationship between the disease outcome and the Neuralgia and smell abnormalities in the studied patients. According to the study, gender is significantly related to headaches caused by central nervous system manifestations in patients. In addition, there is a significant relationship between the disease outcome and the peripheral nervous system and between gender and the peripheral nervous system.

**Table 5:** Determining the relationship between the disease outcome and gender with central and peripheral nervous system manifestations.

Manifestations of the central nervous system		Disease Consequences		Gender	
		Recovery	Death	Male	Female
Dizziness	Yes	42 (2.3%)	1 (0.3%)	21 (1.8%)	22 (2.1%)
	No	1822 (97.7%)	335 (99.7%)	1148 (98.2%)	1009 (99.7%)
The significance level		0.007		0.568	
Headache	Yes	134 (7.2%)	15 (4.5%)	66 (5.6%)	83 (8.1%)
	No	1730 (92.38%)	321 (95.5%)	1103 (94.4%)	948 (91.9%)
The significance level		0.067		0.025	
Impaired consciousness	Yes	21 (1.1%)	32 (9.5%)	33 (2.8%)	20 (1.9%)
	No	1843 (98.9%)	304 (90.5%)	1136 (97.2%)	1011 (98.1%)
The significance level		0.00001		0.178	
Focal neurological symptoms	Yes	4 (0.2%)	2 (0.6%)	2 (0.2%)	4 (0.4%)
	No	1860 (99.8%)	334 (99.4%)	1167 (99.8%)	1027 (99.6%)
The significance level		0.23		0.33	
Stroke	Yes	1 (0.1%)	2 (0.6%)	2 (0.2%)	1 (0.1%)
	No	1863 (99.9%)	334 (99.4%)	1167 (99.8%)	1030 (99.9%)
The significance level		0.063		0.63	
Ataxia	Yes	5 (0.3%)	0 (0%)	3 (0.3%)	2 (0.2%)
	No	1859 (99.7%)	336 (100%)	1166 (99.7%)	1029 (99.8%)
The significance level		0.342		0.75	
convulsions	Yes	3 (0.2%)	4 (1.2%)	2 (0.2%)	5 (0.5%)
	No	1861 (99.8%)	332 (98.8%)	1861 (99.8%)	332 (99.5%)
The significance level		0.002		0.192	

Manifestations of the peripheral nervous system		Disease Consequences		Gender	
		Recovery	Death	Male	Female
Ageusia	Yes	46 (2.5%)	0 (0%)	25 (2.1%)	21 (2%)
	No	1818 (97.5%)	336 (100%)	1144 (97.9%)	1010 (98%)
The significance level		0.004		0.86	
Hyposmia and anosmia	Yes	79 (4.2%)	1 (0.3%)	38 (3.3%)	42 (4.1%)
	No	1785 (95.8%)	335 (99.7%)	1131 (96.7%)	989 (95.9%)
The significance level		0.0001		0.303	
Visual impairment	Yes	3 (0.2%)	0 (0%)	1 (0.1%)	2 (0.2%)
	No	1861 (99.8%)	336 (100%)	1168 (99.9%)	1029 (99.8%)
The significance level		0.608		0.492	
Polyneuropathy	Yes	4 (0.2%)	0 (0%)	2 (0.2%)	2 (0.2%)
	No	1860 (99.8%)	336 (100%)	1167 (99.8%)	1029 (99.8%)
The significance level		0.395		0.90	
Myopathy	Yes	1 (0.2%)	0 (0%)	1 (0.2%)	0 (0%)
	No	1863 (99.8%)	336 (100%)	1168 (99.8%)	1031 (100%)
The significance level		0.67		0.348	

Table 6 demonstrates the correlation between disease outcome with age and blood oxygen level. The *t*‐test shows a statistically significant correlation between the mean age and oxygen level and the disease outcome in the studied patients.

**Table 6:** Determining the relationship between the disease outcome with age and blood oxygen level.

Group		Quantity	Mean	SD	The significance level
Blood oxygen level	Recovery	1864	89.84	6.38	0.0001
	Death	336	78.44	13.15	
Age	Recovery	1864	55.6	16.47	0.0001
	Death	336	67.44	14.44	

Table 7 reveals the relationship between central and peripheral nervous system manifestations with age and blood oxygen level. According to the *t*‐test analysis, a statistically significant difference exists between the mean age and oxygen level in patients with central and peripheral nervous system symptoms (dizziness, headache, impaired consciousness, and impaired smell).

**Table 7:** Determining the relationship between central and peripheral nervous system manifestations with age and blood oxygen level.

Group			Quantity	Mean	SD	The significance level
Dizziness	Blood oxygen level	Yes	43	91.02	6.07	0.003
		No	2157	88.04	8.85	
	Age	Yes	43	49.46	13.7	0.0001
		No	2157	57.57	16.74	
Headache	Blood oxygen level	Yes	149	89.31	7.4	0.043
		No	2051	88.01	8.9	
	Age	Yes	149	52.67	15.34	0.0001
		No	2051	57.75	16.77	
Impaired consciousness	Blood oxygen level	Yes	46	93.36	2.64	0.0001
		No	2154	87.99	8.86	
	Age	Yes	46	35.52	10.58	0.0001
		No	2154	57.87	16.52	
Hyposmia and anosmia	Blood oxygen level	Yes	80	92.71	3.3	0.0001
		No	2120	87.93	8.91	
	Age	Yes	80	37.81	12.38	0.0001
		No	2120	58.15	16.41	

## 7 DISCUSSION

Since COVID‐19 symptoms are similar to flu symptoms, only pulmonary complications are addressed, while nervous system consequences are not. Respiratory failure may occur after the brain stem is infected and the respiratory centers are affected. However, the exact mechanism of COVID‐19 patients' neurological implications is unknown. The peripheral nerves are infected more quickly due to the low level of ACE2 in the central nerve system.^6^, ^20^ The present study aimed to investigate the frequency of neurological symptoms in COVID‐19‐positive patients admitted to the hospital in 2020.

This study's demographic findings showed that the patients' mean age was 57.41 years, and the average blood oxygen level was 88.10%. A total of 53.1% of the studied patients were male, and most disease symptoms were related to shortness of breath and cough (24.3%). In addition, 20.8% of the studied patients needed hospitalization in ICU. The most frequent manifestations of the central and peripheral nervous system were related to headache, dizziness, ageusia, hyposmia, anosmia and respectively. Finally, 15.3% of the studied patients died, and 84.7% recovered. Meanwhile, in Iltaf et al.^21^ headache (6%), altered level of consciousness and encephalopathy (2%), hemiparesis (stroke; 0.6%), Guillain‐Barré Syndrome (0.3%) and seizure (0.3%) were the most frequently reported neurological presentations.^21^ However, Campiglio and Priori (2020) pointed headache, myalgias, altered mental status, ageusia, hyposmia and anosmia^22^ as the most commonly reported symptoms, which was somewhat similar to the result of the present study. Nasrollahzadeh Sabet et al.^23^ examined 1408 patients with COVID‐19 hospitalized in Golestan, Hajar, Khanevadeh, and Besat hospitals in Tehran, Iran, of whom 912 were male (64.8%) and 496 were female (35.2%). The gender ratio was 1.8, and the mean age of the participants was 57.8 years. The age range of all patients varied from 18 to 98 years. Intubations were performed on 27% of all patients, and 21.9% died. All patients were divided into two groups based on their blood oxygen levels, of whom 53.7% had a level under 93%, while 46.3% had a level above 93%.^23^ Ling et al. (2020) evaluated 214 patients in China, of whom 88 (41.1%) were severe and 126 (58.9%) were nonsevere.

A total of 78 patients (36.4%) had neurological manifestations. The most common neurologic symptoms in severe COVID‐19 patients were acute brain disease, impaired consciousness, and skeletal muscle symptoms.^24^ Leonardi et al.^20^ studied neurological manifestations associated with COVID‐19, during which a systematic review of articles was published up to April 5, 2020. A review of 29 articles related to neurological manifestations of COVID‐19 was conducted. The results indicated the presence of central and peripheral nervous system manifestations related to COVID‐19 and stated that neurological manifestations or neurocovid are part of COVID‐19.^20^ The findings of this study showed a significant relationship between the disease outcome with dizziness, impaired consciousness, and seizures. A higher percentage of patients with dizziness and headache recovered, while a higher rate of patients with consciousness disorders and seizures died.

Studies of the damage caused by COVID‐19 on the respiratory system have shown that the efficiency of this system for the exchange of respiratory gases decreases, and the amount of oxygen in the blood decreases, which can lead to impaired brain function and a decrease in the level of consciousness and convulsions.^25^, ^26^ In addition, there is a significant relationship between the disease outcome and ageusia, hyposmia and anosmia in the studied patients. The highest percentage of patients with these disorders improved, and only one died from a smell disorder. Ageusia, hyposmia and anosmia are the most common symptoms of the effect of COVID‐19 on the nervous system in patients with COVID‐19.^27^ Unlike other systems, the nerve receptors of the olfactory system are located precisely on the nerve. When the virus can infect the receptor, the nerve can be infected with the virus, entering directly from this point and involving brain neurons.^28^, ^29^

The present study evaluated the relationship between gender, disease outcome, and central and peripheral nervous system manifestations. The results showed a significant relationship between gender and headache, revealing that females had the highest number of headaches. According to the study, there is a statistically significant association between the mean age and oxygen level with symptoms of the central and peripheral nervous systems (dizziness, headache, impaired consciousness, impaired smell) and the disease outcome. People who died had a lower average blood oxygen level and an older mean age. Hokkaido University in Japan found that people's age is not influential in the possibility of contracting COVID‐19. Symptom severity, disease progression, and death rates are affected by the patient's age of.^30^

This study had several limitations, including its retrospective design, which prevented consideration of confounding factors. Moreover, a series of information related to the variables were not registered and collected by contacting the hospitalized people. Many patients died, and the first‐degree relatives of the patients were obtained verbally to use the patients' information in the informed consent study. However, the strength of this study is the large number of samples since this hospital was the only center where COVID‐19 patients were hospitalized.

Patients were never contacted during the entire duration of the study.

The limitations of the current study were the nonrandomized methodology and the possibility of bias due to the inherent nature of observational, open‐label *research*.

Studying variables such as drug use history and underlying diseases over a long period is recommended for future research. Irrespective of the mechanism, neurological manifestations should be monitored in COVID‐19 patients carefully. An important matter will be to investigate whether neurological disturbances impact the prognosis of COVID‐19.

## 8 CONCLUSION

The pathophysiology of COVID‐19 virus infection involving the central nervous system is not fully understood. Neurological symptoms of this virus include delirium, headache, decreased level of consciousness, and seizures. Understanding the symptoms and mechanisms of neurological complications of COVID‐19 is necessary for proper screening and complete treatment of the infected because an infected patient may not show respiratory failure signs but may be a carrier. Therefore, thorough knowledge of the symptoms and complications of infection with this virus is necessary to screen patients and adequately prevent the spread of this disease. The virus spreads to the central nervous system in direct and indirect ways. Neurological manifestations of COVID‐19 are still unknown because many cases of this disease are also misdiagnosed as other febrile illnesses. Therefore, neurological manifestations of COVID‐19 should be considered in the differential diagnosis of patients with these neurological signs and symptoms.

## AUTHOR CONTRIBUTIONS

**Shima Rasouli**: Data curation; investigation; project administration; resources; validation; writing—review and editing. **Payam Emami**: Formal analysis; investigation; writing—original draft; writing—review and editing. **Farhad Azadmehr**: Investigation; software; supervision; visualization; writing—review and editing. **Farzaneh Karimyan**: Conceptualization; funding acquisition; project administration; supervision; visualization; writing—original draft; writing—review and editing.

## CONFLICT OF INTEREST STATEMENT

The authors declare no conflict of interest.

## ETHICS STATEMENT

The Ethics Committees of the Kurdistan University of Medical Sciences, Iran (IR.MUK.REC.1399.288) approved the study protocol. Alive patients were informed of the research objectives, anonymous responses, and confidentiality terms regarding their information.

## TRANSPARENCY STATEMENT

The lead author Farzaneh Karimyan affirms that this manuscript is an honest, accurate, and transparent account of the study being reported; that no important aspects of the study have been omitted; and that any discrepancies from the study as planned (and, if relevant, registered) have been explained.

## Supporting information

Supporting information.

## Data Availability

The data that support the finding of this study are available on request from the corresponding author. The data are not publicly available due to privacy or ethical restrictions.

## ENDNOTE

* Intensive care unit.

## References

[1] Collantes MEV , Espiritu AI , Sy MCC , Anlacan VMM , Jamora RDG . Neurological manifestations in COVID‐19 infection: a systematic review and meta‐analysis. Can J Neurol Sci. 2021;48(1):66‐76. 32665054 10.1017/cjn.2020.146 PMC7492583

[2] Lahiri D , Ardila A . COVID‐19 pandemic: a neurological perspective. Cureus. 2020;12(4). 10.7759/cureus.7889 PMC7255551 32489743

[3] Zhang C , Shi L , Wang F‐S . Liver injury in COVID‐19: management and challenges. Lancet Gastroenterol Hepatol. 2020;5(5):428‐430. 32145190 10.1016/S2468-1253(20)30057-1 PMC7129165

[4] Ali ST , Kang AK , Patel TR , et al. Evolution of neurologic symptoms in non‐hospitalized COVID‐19 “long haulers”. Ann Clin Transl Neurol. 2022;9(7):950‐961. 35607826 10.1002/acn3.51570 PMC9268866

[5] World Health Organization . (2020). Responding to community spread of COVID-19: interim guidance, 7 March 2020. World Health Organization.

[6] Lauer SA , Grantz KH , Bi Q , et al. The incubation period of coronavirus disease 2019 (COVID‐19) from publicly reported confirmed cases: estimation and application. Ann Intern Med. 2020;172(9):577‐582. 32150748 10.7326/M20-0504 PMC7081172

[7] Backer JA , Klinkenberg D , Wallinga J . Incubation period of 2019 novel coronavirus (2019‐nCoV) infections among travellers from Wuhan, China, 20–28 January 2020. Euro Surveill. 2020;25(5):2000062. 32046819 10.2807/1560-7917.ES.2020.25.5.2000062 PMC7014672

[8] Vitalakumar D , Sharma A , Kumar A , Flora S . Neurological manifestations in COVID‐19 patients: a meta‐analysis. ACS Chem Neurosci. 2021;12(15):2776‐2797. 34260855 10.1021/acschemneuro.1c00353

[9] Bialek S , Gierke R , Hughes M , McNamara LA , Pilishvili T , Skoff T . Coronavirus disease 2019 in children—United States, February 12–April 2, 2020. MMWR Morb Mortal Wkly Rep. 2020;69(14):422‐426. 32271728 10.15585/mmwr.mm6914e4 PMC7147903

[10] Guo Y‐R , Cao Q‐D , Hong Z‐S , et al. The origin, transmission and clinical therapies on coronavirus disease 2019 (COVID‐19) outbreak–an update on the status. Mil Med Res. 2020;7(1):11. 32169119 10.1186/s40779-020-00240-0 PMC7068984

[11] Pavone P , Ceccarelli M , Marino S , et al. SARS‐CoV‐2 related paediatric acute‐onset neuropsychiatric syndrome. Lancet Child Adol Health. 2021;5(6):19. 10.1016/S2352-4642(21)00135-8 PMC8096321 33961798

[12] Ahmed MU , Hanif M , Ali MJ , et al. Neurological manifestations of COVID‐19 (SARS‐CoV‐2): a review. Front Neurol. 2020;11:518. 32574248 10.3389/fneur.2020.00518 PMC7257377

[13] Iadecola C , Anrather J , Kamel H . Effects of COVID‐19 on the nervous system. Cell. 2020;183(1):16‐27. 32882182 10.1016/j.cell.2020.08.028 PMC7437501

[14] Guan W , Ni Z , Hu Y , et al. Clinical characteristics of coronavirus disease. NEJMoa2002032. 2020;58(4):711‐712.

[15] Favas TT , Dev P , Chaurasia RN , et al. Neurological manifestations of COVID‐19: a systematic review and meta‐analysis of proportions. Neurol Sci. 2020;41:3437‐3470. 33089477 10.1007/s10072-020-04801-y PMC7577367

[16] Durjoy L , Alfredo A . COVID‐19 pandemic: a neurological perspective. Cureus. 2020;12(4):e7889. 32489743 10.7759/cureus.7889 PMC7255551

[17] Nikbakht F , Mohammadkhanizadeh A , Mohammadi E . How does the COVID‐19 cause seizure and epilepsy in patients? the potential mechanisms. Multi Sclero Rela Disor. 2020;46:102535. 10.1016/j.msard.2020.102535 PMC7521932 33010584

[18] Xu E , Xie Y , Al‐Aly Z . Long‐term neurologic outcomes of COVID‐19. Nature Med. 2022;28(11):2406‐2415. 36138154 10.1038/s41591-022-02001-z PMC9671811

[19] Shakiba M , Nazemipour M , Heidarzadeh A , Mansournia MA . Prevalence of asymptomatic COVID‐19 infection using a seroepidemiological survey. Epidemiol Infect. 2020;148:300. 10.1017/S0950268820002745 PMC7783089 33183367

[20] Leonardi M , Padovani A , McArthur JC . Neurological manifestations associated with COVID‐19: a review and a call for action. J Neurol. 2020;267(6):1573‐1576. 32436101 10.1007/s00415-020-09896-z PMC7238392

[21] Iltaf Sr. S , Fatima M , Salman Sr. S , Salam J‐u , Abbas S . Frequency of neurological presentations of coronavirus disease in patients presenting to a tertiary care hospital during the 2019 coronavirus disease pandemic. Cureus. 2020;12(8):e9846. 32953353 10.7759/cureus.9846 PMC7497771

[22] Campiglio L , Priori A . Neurological symptoms in acute COVID‐19 infected patients: a survey among Italian physicians. PLoS One. 2020;15(9):e0238159. 32915818 10.1371/journal.pone.0238159 PMC7489055

[23] Nasrollahzadeh Sabet M , Khanalipour M , Gholami M , Sarli A , Rahimi Khorrami A , Esmaeilzadeh E . Prevalence, clinical manifestation and mortality rate in COVID‐19 patients with underlying diseases. J Arak University Med Sci. 2020;23(5):740‐749.

[24] Mao L , Wang M , Chen S , et al. Neurological manifestations of hospitalized patients with COVID‐19 in wuhan, China: a retrospective case series study. MedRxiv. 2020;77(6):683‐690. 10.1001/jamaneurol.2020.1127 PMC7149362 32275288

[25] Lovato A , De Filippis C . Clinical presentation of COVID‐19: a systematic review focusing on upper airway symptoms. Ear Nose Throat J. 2020;99(9):569‐576. 32283980 10.1177/0145561320920762

[26] Karimi N , Sharifi Razavi A , Rouhani N . Frequent convulsive seizures in an adult patient with COVID‐19: a case report. Iran Red Crescent Med J. 2020;22(3):e102828.

[27] Su S , Wong G , Shi W , et al. Epidemiology, genetic recombination, and pathogenesis of coronaviruses. TIM. 2016;24(6):490‐502. 10.1016/j.tim.2016.03.003 PMC7125511 27012512

[28] Wu F , Zhao S , Yu B , et al. A new coronavirus associated with human respiratory disease in China. Nature. 2020;579(7798):265‐269. 32015508 10.1038/s41586-020-2008-3 PMC7094943

[29] Ahmad I , Rathore FA . Neurological manifestations and complications of COVID‐19: a literature review. J Clin Neurosci. 2020;77:8‐12. 32409215 10.1016/j.jocn.2020.05.017 PMC7200361

[30] Omori R , Matsuyama R , Nakata Y The age distribution of mortality from novel coronavirus disease (COVID‐19) suggests no large difference of susceptibility by age. Sci Rep 2020;10(1):16642. 33024235 10.1038/s41598-020-73777-8 PMC7538918

