# Supplementary material for: Evaluating the frequency of neurological symptoms in COVID‐19 patients: A cross‐sectional study
Source: Health Sci Rep. 2023 Jul 21;6(7):e1400. doi: 10.1002/hsr2.1400 (PMC10363788; doi:10.1002/hsr2.1400)
Supplement: Supplementary file 1 — Supporting information. [file HSR2-6-e1400-s001.docx]

| **Copyright Transfer Statement**   \|  \| **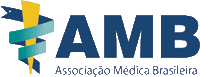**  **RAMB -** Revista da Associação Médica Brasileira \| \| --- \| --- \| \| Flux Code: \|  \| \| Title: \| **Evaluating the Frequency of Neurological Symptoms in COVID-19 Patients: A Cross-Sectional Study** \|   The author(s) of the article as specified herein, hereby transfer copyright and assigns to Revista da Associação Médica Brasileira (RAMB) all rights, title and interest that the author may have, or may at any time be found to have in and to the article and any revisions or versions thereof, including, but not limited to, the sole right to print, publish, and sell the article throughout the world in all languages and media.  This assignement shall be deemed in effect if and when the article is accepted for publication.  Should the article contain any material protected by the copyright of others, the author will deliver to RAMB written permission from the copyright owner to reproduce such material in the article. The(is) author(s) represents and warrants the are author(s) and proprietor of the article, that are has not granted or assigned any rights in the article to any other person or entity, that the article is copyrightable, that is does not infringe upon any copyright, trademark, or patent, that it does not invade the right of privacy or publicity of any person or entity, that it does not contain any libelous matter, that all statements asserted as facts are true or based upon reasonable research to accuracy and that, to the best of the author's knowledge, no formula, procedure, or prescription contained in the article would cause injury if used or followed in accordance with instructions and/or warnings contained in the article.  The author(s) will indemnity RAMB against any costs, expenses or damages that RAMB may incur or for which RAMB may become liable as a result of any breach of these warranties. These representations and warranties may be extended to third parties by RAMB. |
| --- | --- | --- | --- | --- | --- | --- |
|  |
|  |
|  |
| **Does your article include material from other copyrighted sources?   Yes  No** (If yes, please attach relevant permissions)  **Does your article include illustrations in which a person can be recognized?   Yes  No**(if yes, please attach relevant permissions) |

Date:

Author (s) signature___________Shima Rasouli______
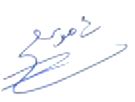


Author (s) signature___________Payam Emami ____________
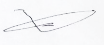


Author (s) signature___________Farhad Azadmehr _________
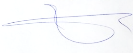


Author (s) signature___________Farzaneh Karimyan ________
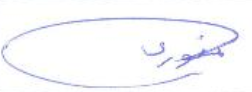


**Please, send to e-mail ramb@amb.org.br**
